# Rapid and Specific Detection of the Poplar Black Spot Disease Caused by *Marssonina brunnea* Using Loop-Mediated Isothermal Amplification Assay

**DOI:** 10.3390/plants10020253

**Published:** 2021-01-28

**Authors:** Qin Xiong, Linlin Zhang, Xinyue Zheng, Yulin Qian, Yaxin Zhang, Lijuan Zhao, Qiang Cheng

**Affiliations:** 1 Co-Innovation Center for Sustainable Forestry in Southern China, College of Biology and the Environment, Nanjing Forestry University, Nanjing 210037, China; xiongqin@njfu.edu.cn (Q.X.); lindazllxs@163.com (L.Z.); zhengxinyue5806@126.com (X.Z.); qly_1998@126.com (Y.Q.); zyx2545382526@163.com (Y.Z.); 2 The Southern Modern Forestry Collaborative Innovation Center, Nanjing Forestry University, Nanjing 210037, China; zhaolijuan@njfu.edu.cn

**Keywords:** *Marssonina brunnea*, rapid detection, loop-mediated isothermal amplification, poplar black spot

## Abstract

*Marssonina brunnea* is the main pathogen that causes poplar black spot disease, which leads to the decrease of the photosynthetic efficiency and significantly affects the production and quality of timber. Currently, no in-field diagnostic exists for *M. brunnea*. Here, we described a loop-mediated isothermal amplification (LAMP) assay for the rapid and sensitive detection of *M. brunnea*. A set of six oligonucleotide primers was designed to recognize eight distinct sequences of the internal transcribed spacer (*ITS*) region of *M. brunnea*. The LAMP assay was optimized by the combination of high specificity, sensitivity, and rapidity for the detection of less than 10 pg/μL of target genomic DNA in 60 min per reaction at 65 °C, whereas with PCR, there was no amplification of DNA with concentration less than 1 ng/μL. Among the genomic DNA of 20 fungalisolates, only the samples containing the genomic DNA of *M. brunnea* changed from violet to sky blue (visible to the naked eye) by using hydroxynaphthol blue (HNB) dye. No DNA was amplified from the eight other fungus species, including two other *Marssonina* pathogens, three other foliar fungi pathogens of poplar, and three common foliar fungal endophytes of poplar. Moreover, the detection rates of *M. brunnea* from artificially and naturally infected poplar leaves were 10/16 (62.5%) and 6/16 (37.5%) using PCR, respectively, while the positive-sample ratios were both 16/16 (100%) using the LAMP assay. Overall, the *ITS* LAMP assay established here can be a better alternative to PCR-based techniques for the specific and sensitive detection of *M. brunnea* in poplar endemic areas with resource-limited settings.

## 1. Introduction

The poplar black spot disease is one of the most destructive foliar diseases that often severely breaks out in poplar plantations all over the world recently [1,2,3,4,5,6]. The fungal species *Marssonina brunnea* (Ellis & Everh.) Magnus is the most pathogenic species, attacking species of the three largest and commercially most important sections of the genus *Populus*, i.e., Tacamahaca, Aigieros, and Leuce, causing premature defoliation and, ultimately, weakening and dieback of the tree [6,7,8]. On the basis of the host range and conidial morphology, research has classified the population of *M. brunnea* into two specialized forms, *M. brunnea* f. sp. *multigermtubi* and *M. brunnea* f. sp. *monogermtubi* [9]. The former is hosted on many species of section of Aigieros and their hybrids, as well as hybrids of sections Aigieros and Tacamahaca. The conidia germinate 1–5 (generally 2–3) germtubes, whereas the latter is only pathogenic to poplars of the section Leuce, such as *Populus adenopoda* and *Populus tomentosa*, and their hybrids. The conidia germinate one germtube [8,9]. The germtubes penetrated poplar leaves directly through the cuticle and epidermal wall, and indirectly through stomata without forming appressoria [10]. Ultimately, characteristic fruiting bodies (acervuli) are formed on leaf and shoot lesions and developing conidia in epidermal cells. Masses of conidia released from these acervuli initiate secondary infections in the developing tree canopy [2].

In China, *Marssonina* leaf spot disease is present in most of the poplar-growing regions, such as Shandong, Jiangsu, Henan, Shanxi, and Jilin provinces, as well as Beijing [11]. The punctiform spots caused by *M. brunnea* darken with age and gradually coalesce to form angular, circular, or lens-shaped necrotic blotches with a yellow to golden margin [6]. The fungus causes these spots on the poplar leaves, petioles, and sometimes on young green twigs and capsules [12], leading to serious economic losses. Under favorable conditions, the disease can develop rapidly and cause early leaf fall in August. In particular, the symptoms in the early stage are difficultly discerned with naked eye and are likely to be confused with other poplar leaf disease, such as poplar leaf and shoot blight caused by *Venturia populina* (Vuill.) Fabric. [13] and poplar spot anthracnose caused by *Elsinoë australis* [14]. Moreover, the symptoms might differ significantly from one poplar variety to another.

Due to the continuing shrinkage of natural forests, hybrid poplars (*Populus* spp.) with exceptional growth rates have been increasingly planted worldwide in a short rotation intensive culture, aimed to maximize carbon sequestration, and fiber and woody biomass production [15,16]. However, the infection of *Marssonina* leaf spot severely reduces the growth and productivity of hybrid poplars. Infestations may be more severe from one year to the next. As a consequence of this situation, efficient diagnostic tools are needed for the monitoring of this pathogen as early as possible in symptomless plants. To our knowledge, no investigators have reported any detection systems for rapid detection of *M. brunnea,* including the nucleic acid amplification method. Up to now, only three *Marssonina* species, namely, *M. brunnea* f. sp. *multigermtubi* (https://mycocosm.jgi.doe.gov/Marbr1/Marbr1.home.html), *M. brunnea* f. sp. *monogermtubi* (https://mycocosm.jgi.doe.gov/Mabrmo1/Mabrmo1.home.html), and *Marssonina coronaria* (Ellis & Davis) Davis [National Center for Biotechnology Information (NCBI) Genbank ASM220425v1], have sequenced the complete genome.

Advanced molecular techniques, such as polymerase chain reaction (PCR), nested-PCR, real-time PCR, and DNA microarray, have been successfully used to detect a broad range of plant pathogens [17,18,19]. However, these methods require specialized personnel, sophisticated equipment, and expensive reagents, which generally are unavailable or difficult or costly to obtain in developed and underdeveloped countries. Thus, there is a need for alternative, easy, rapid, and low-cost techniques that require only a few reagents and basic laboratory equipment. Loop-mediated isothermal amplification (LAMP) is an unconventional method of DNA amplification developed by Notomi et al. [20], which does not need the thermal cycling of PCR and can be conducted in an instrument that can maintain constant temperature, such as a water bath or heat block. In addition, the LAMP polymerase enzyme is less sensitive to inhibiting substances than those used for PCR reactions, allowing simpler extraction method of crude DNA to be used [21,22]. In contrast to PCR-based methods, the LAMP assay has the great advantages of real-time detection, visual inspection, high amplification efficiency, and high degree of sensitivity and specificity [23,24,25]. It is easily adapted for on-site detection of plant pathogens under field conditions [18].

Due to these advantages, LAMP assays have been commonly used for rapid diagnosis of plant pathogenic viruses [26], fungi [27,28,29], bacteria [30,31], oomycete [32,33,34,35], and parasite [36], including several invasive tree pathogens [35,37,38]. In this study, we aimed to develop a LAMP assay for the detection of *M. brunnea* using a species-specific target internal transcribed spacer (*ITS*) sequence, as well as to evaluate its efficacy with PCR for the early diagnosis of *M. brunnea* in fields. This is the first report to develop a rapid and field deployable detection assay of *M. brunnea.*

## 2. Results

### 2.1. Primers Required for LAMP Reaction

A successful LAMP reaction with species-specific primers produced many bands of different sizes in a reproducible ladder-like pattern. When the sample tube did not contain selected target DNA or any of the primers F3 (forward outer primer), B3 (backward outer primer), FIP (forward inner primer), and BIP (backward inner primer), no amplification was observed.

**Figure 1:**
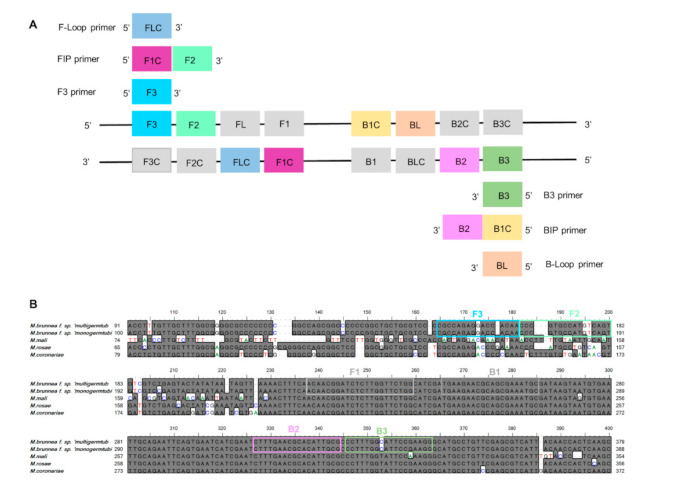
Design of LAMP primers specific for *Marssonina brunnea* based on internal transcribed spacer (*ITS*) sequences. (**A**) Schematic representation of LAMP-amplified regions. For ease of explanation, six distinct regions are designated on the target DNA, labeled F3 (forward outer primer), F2, F1, B1C, B2C, and B3 (backward outer primer) from the 5’ end. As C represents a complementary sequence, F1C and B1C are complementary to F1 and B1, respectively. Two inner primers (FIP (forward inner primer) and BIP (backward inner primer)) and outer primers (F3 and B3) are used in the LAMP method. FIP (BIP) is a hybrid primer consisting of the F1C (B1C) sequence and the F2 (B2) sequence. FL and BL represent forward and backward loop primers, respectively. (**B**) Nucleotide sequence alignment of *ITS* sequences from *M. brunnea* f. sp. *multigermtubi*, *M. brunnea* f. sp. *monogermtubi*, and three closely related species. In the aligned sequences, grey shade indicates a match, and a dash indicates a gap sequence. Polymorphic sites among the five species are highlighted in color. The rectangular boxes with different colors correspond to the different primer sequences of the LAMP primer set shown in A.

In testing of all primers using the nucleotide Basic Local Alignment Search Tool (BLAST) on the NCBI sequence database revealed no significant hits as to August 2020, suggesting high primer specificity for the target sequences. Moreover, the specificity of the *M. brunnea ITS* amplicons amplified with F3 and B3 primers (Table 1) was checked against the NCBI Nucleotide database using nucleotide BLAST (BLASTn) analysis, which showed a 100% identity with sequences of *Marssonina brunnea* f. sp. *multigermtubi* strain HY14 and *Marssonina brunnea* f. sp. *monogermtubi* strain BBHB2 (GenBank accession numbers KU508806 and KM246324, respectively) (Figure 1B).

### 2.2. Optimization of LAMP Assay Conditions

**Figure 2:**
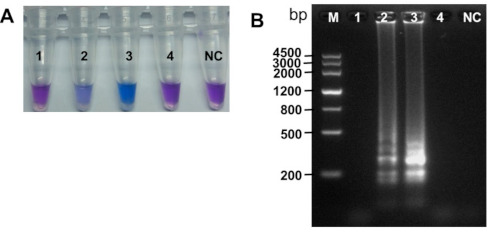
Effects of different reaction temperatures on efficiency and specificity of loop-mediated isothermal amplification (LAMP) amplification. (**A**) Visualization by hydroxynaphthol blue (HNB) in amplification tubes. Sky blue color indicates positive DNA amplification of *Marssonina brunnea* (Tubes 2 and 3). (**B**) The 2% agarose gel showing products from LAMP reaction. Ladder-like pattern indicates positive reactions (lanes 2 and 3). M, molecular size ladder; tubes and lanes 1–4, LAMP reactions carried out with 100 pg genomic DNA of *M. brunnea* f. sp. *multigermtubi* strain XY-3 at different incubation temperatures (60, 62.5, 65.0, 67.5 °C, respectively); NC = negative control (no DNA).

To determine the optimum temperature for efficient amplification by the LAMP assay, we incubated the LAMP reaction mixtures at 60, 62.5, 65, and 67.5 °C for 60 min, using the four basic LAMP primers (F3, B3, FIP, and BIP). A total of 100 pg of genomic DNA of *M. brunnea* f. sp. *multigermtubi* strain XY-3 was selected as a template. The results are shown in Figure 2A,B. The clear DNA ladder-like pattern and color change only appeared at 62.5 °C and 65.0 °C. Nonspecific reactions were not observed at other reaction temperatures (Figure 2A,B). Because higher reaction temperatures decrease nonspecific primer annealing [39], and the amplification products displayed a much clearer DNA ladder-like pattern and color change from violet to sky blue at 65.0 °C than at 62.5 °C, we selected 65.0 °C for further evaluation. Following standardization, optimization of the LAMP assay was also carried out with regard to the effect of two additional loop primers on reaction kinetics and sensitivity. We found that the positive LAMP results (violet-to-blue change and typical DNA ladder-like pattern) could be observed after only 35 min when the forward and backward loop primers were added. Therefore, we used the loop primers in the following tests to accelerate the amplification reaction.

### 2.3. Specificity of the LAMP Assay

**Figure 3:**
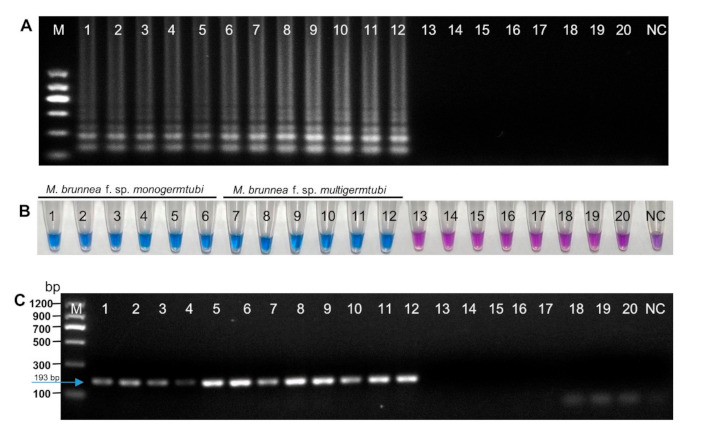
Specific detection of *M. brunnea* by the LAMP and PCR assays. (**A**) LAMP assay products visualized by agarose gel electrophoresis. (**B**) LAMP assay products visualized by hydroxynaphthol blue (HNB). (**C**) PCR assay products on agarose gel. Lanes and tubes 1–6 are *M. brunnea* f. sp. *monogermtubi* strains (QC2, QM2, QM3, QM6, QM8, and QM15, respectively); lanes and tubes 7–12 are *M. brunnea* f. sp. *multigermtubi* strains (J1, J3, 214-2, 214-4, XY-1, and XY-3, respectively); and lanes and tubes 13–20 are other tested fungi (*M. coronaria* Mp-1, *M. rosae* Ms-3, *Melampsora larici-populina* YL-1, *Venturia populina* T4, *Elsinoë australis* NL-1*, Alternaria* sp. As-1, *Botryosphaeria* sp. Bs-1, and *Pseudocercospora* sp. Ps-1, respectively). NC = negative control (no DNA); M = molecular size ladder.

The specificity of the LAMP assay was determined with DNA from 12 *M. brunnea* isolates*,* 2 isolates of two other *Marssonina* species, and 6 isolates of other poplar foliar fungi including three common foliar fungal endophytes of poplar (*Alternaria* sp., *Botryosphaeria* sp., and *Pseudocercospora* sp.) and three other foliar pathogens of poplar (*Melampsora larici-populina* Kleb., *Venturia populina* (Vuill.) Fabr., and *Elsinoë australis*) (Table 2). Positive or negative results were confirmed by adding hydroxynaphthol blue (HNB) to the reaction system prior to amplification. After the reaction, the color of the positive samples changed to sky blue, differing from the violet color in the negative samples. Meanwhile, the LAMP amplification products of the positive samples displayed the typical ladder-like patterns in agarose gel electrophoresis. As shown in Figure 3, 12 *M. brunnea* strains, whether isolated from *Populus tomentosa* Carr., *Populus × canadensis* Moench, and *Populus × euramericana* “I-214” in Jiangsu Province, or isolated from *Populus simonii* Carr. in Liaoning Province, showed positive LAMP reactions, whereas reactions containing two other *Marssonina* species (*Marssonina coronaria* and *Marssonina rosae*) and six other fungal species were negative (Figure 3A,B). At least three replicates were analyzed to verify the specificity of the LAMP reactions. PCRs were performed using the same DNA samples and the same result was observed. Amplification products of 193 bp were only observed in the tubes containing *M. brunnea* DNA as compared to no products in negative control and other fungi (Figure 3C). This was indicative of the ribosomal DNA internal transcribed spacer (rDNA-ITS) region being suitable as a target for detecting and identifying *M. brunnea*.

### 2.4. Sensitivity of the LAMP and PCR Assays

**Figure 4:**
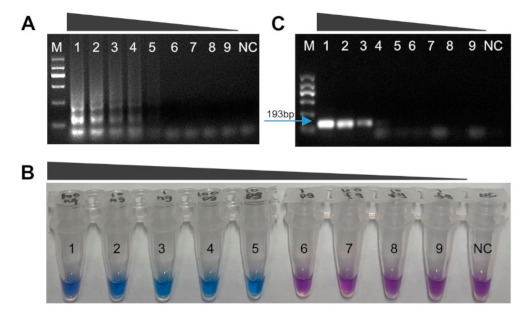
Sensitivity of the LAMP and PCR assays for the detection of *M. brunnea*. (**A**) LAMP assay products visualized by agarose gel electrophoresis. (**B**) LAMP assay products visualization by hydroxynaphthol blue (HNB). (**C**) PCR assay products on agarose gel. M = molecular size ladder. Lanes and tubes 1–9 are template DNA concentrations (100 ng/μL, 10 ng/μL, 1 ng/μL, 100 pg/μL, 10 pg/μL, 1 pg/μL, 100 fg/μL, 10 fg/μL, and 1 fg/μL, respectively); NC = negative control (no DNA).

To determine the sensitivity of the LAMP assay, we performed reactions using 10-fold serial dilutions of a 100 ng/μL genomic DNA solution (*M. brunnea* strain XY-3) down to 1 fg/μL. The results are shown in Figure 4A,B, in which the DNA concentrations decreased from left to right. The minimum concentration detected was 10 pg/μL in the LAMP assay (Figure 4A,B). PCRs were performed using the same DNA samples. PCR amplification products of 193 bp were observed in samples ranging from 100 ng/μL to 1 ng/μL (Figure 4C). Negative control showed no amplification under the same conditions (Figure 4A–C). The LAMP and PCR assays were carried out in triplicate for each template DNA. The results suggest that the LAMP assay (10 pg/μL) has a 100-fold greater sensitivity as compared with conventional PCR (1 ng/μL).

### 2.5. Direct Detection of M. brunnea in Artificially Inoculated Poplar Samples

**Figure 5:**
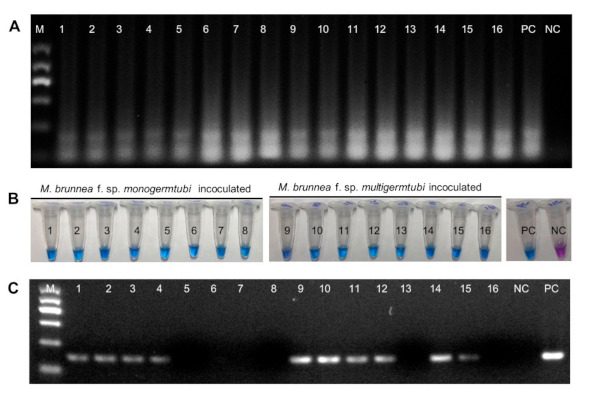
Detection of *M. brunnea* from artificially infected poplar leave samples by the LAMP and PCR assays. (**A**) Detection *M. brunnea* in artificial infected poplar leaves by LAMP assay conducted on the basis of the appearance of a ladder-like pattern. Negative reactions did not show any ladder-like patterns. (**B**) Detection *M. brunnea* in artificial infected poplar leaves by LAMP assay conducted on the basis of color change. PC = positive control and NC = negative control. The positive reactions (infected leaves) showed a color change to sky blue while negative amplification reactions remained violet in color. (**C**) Detection *M. brunnea* in artificial infected poplar leaves by PCR assay. PC = positive control [purified genomic DNA (gDNA) of *M. brunnea* strain XY-3]; NC = negative control (purified gDNA from *M. brunnea*-free poplar leaves spray-inoculated with deionized water liquid). Lanes and tubes 1–8 are purified gDNA from poplar leaves infected by *M. brunnea* f. sp. *monogermtubi* strain QC2; lanes and tubes 9–16 are purified gDNA from poplar leaves infected by *M. brunnea* f. sp. *multigermtubi* strain 214-4; M = molecular size ladder.

Artificially inoculated poplar leaves exhibiting symptoms of black spots in the greenhouse were evaluated by the LAMP and PCR assays. The positive-sample ratios were 16/16 (100%) by the LAMP assay (Figure 5A,B) and 10/16 (62.5%) by conventional PCR (Figure 5C). By contrast, negative results were obtained in healthy leaves inoculated with liquid water and no DNA sample by both PCR method and LAMP reaction (Figure 5). The results showed that the LAMP assay succeeded in detecting *M. brunnea* in the infected poplar samples.

### 2.6. Direct Detection of M. brunnea in Diseased Poplar Leaves from the Field

**Figure 6:**
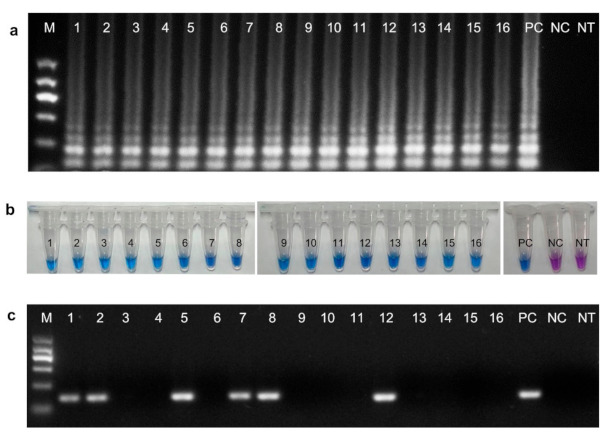
Detection of poplar black spot pathogen in the field samples by the LAMP and PCR assays. (**a**) LAMP assay products visualized by agarose gel electrophoresis. (**b**) LAMP assay products visualization by hydroxynaphthol blue (HNB). (**c**) PCR assay. PC = positive control (purified gDNA of *M. brunnea* strain XY-3); NC = negative control (purified gDNA from *M. brunnea*-free poplar leaves); NT = no DNA template. Lanes and tubes 1–16 are purified gDNA extracted from 16 leaves exhibiting classical black spot symptoms; M = molecular size ladder.

The LAMP assay for *M. brunnea* detection was applied to the diseased poplar leaves collected in the field in the campus of Nanjing Forestry University. As shown in Figure 6, the presence of *M. brunnea* could be accurately detected in a total of 16 diseased leaf samples by the LAMP assay (Figure 6A,B), while *M. brunnea* was detected in only 6 out of 16 diseased leaf samples by PCR (Figure 6C). No amplification products were observed in the negative control (*M. brunnea*-free leave sample) or no template (NT) control. These results were consistent between two independent repeats of each assay. These results indicate that the LAMP assay reported here could be successfully applied to field-collected poplars to detect the poplar black pot disease.

## 3. Discussion

This work successfully established and optimized a rapid and specific diagnostic LAMP assay conducted on the basis of rDNA *ITS* region of the forest pathogen *M. brunnea*. Owing to the unavailable of the complete genome sequences or other traditional genetic markers (i.e., β-tubulin gene, histone H3 gene, and calmodulin gene) of all *Marssonina* pathogens mentioned in this study, the only choice of the target selection is the rDNA *ITS* gene. *Marssonina coronaria*, a devastating plant pathogen causing apple blotch, possesses different detection methods, including optical coherence tomography (OCT)-based technique, hyperspectral imaging system, PCR, quantitative PCR (Q-PCR), and LAMP [40,41,42,43,44]. In the literature to date, only the rDNA *ITS* sequence of *M. coronaria* has been selected as the target for primer design. The sequence alignment of the *ITS* region has shown 95 to 100% homology among the different strains of *Marssonina* from diverse geographical locations (Figure 1B). After considering several key factors for designing LAMP primers, such as primer length, GC content, stability, primer secondary structures, melting temperature (Tm), and the distance between primers [45], we selected one primer set that satisfied all design guidelines, but it showed B3 is only one bp differ from the others, and that F1, B1, and B2 are the same as the other three closely related species. However, the primer set surprisingly had the ability to distinguish the closely related species from each other, suggesting that F3 and F2 primers may play essential roles in avoiding cross homologies during LAMP detection assay of *M. brunnea*. In the future, maybe the other potential targets for molecular diagnostics could be identified by bioinformatics tools, such as integrated or comparative genomic, transcriptomic, and proteomic approaches. Dai et al. [37] recently applied comparative genomics approaches for the design of LAMP detection assays of *Phytophthora cinnamomi*. 

In this study, the comparative evaluation of LAMP vs. conventional PCR revealed LAMP stands out a preferred diagnostic tool with higher sensitivity, specificity, rapidity, simplicity, and adaptability to field conditions. The LAMP assay was found to be 100-fold more sensitive than PCR with a minimum detection limit of 10 pg/μL template (Figure 4). Further, the sensitivity can be significantly improved by multiple endonuclease restriction real-time-LAMP technology [46], dye-binding Q-PCR [47], or biosensor assays, making the LAMP assay even more efficient at detecting *M. brunnea* in poplar. Therefore, it could be a better alternative method for screening samples with minute quantities of *M. brunnea* that might escape the PCR detection. Moreover, it was markedly faster compared with PCR, requiring just 35 min to complete the reaction rather than at least 90 min for PCR. The high amplification efficiency of LAMP is attributed to the lack of a need to choose complex-variable temperature conditions and set different response procedures [25]. That is to say, the LAMP has great advantage over PCR in terms of rapidity, wherein LAMP allows for immediate diagnosis. 

The LAMP assay also demonstrated a higher degree of specificity for detection of *M. brunnea* in both the artificially inoculated and naturally infested poplar leaves, having no false-positive or false-negative results when compared with PCR assay (Figure 5 and Figure 6), indicating that it is highly specific for the target sequence. Specificity of the LAMP and PCR-based assays were also validated by the negative detection results against eight isolates belonging to other species, including two other reported *Marssonina* pathogens, three common foliar fungal endophytes of poplar, and three other foliar pathogens of poplar with same spot symptom in the early stage as that caused by *M. brunnea*. Due to the limitation of the currently reported *Marssonina* pathogens, only two well-known isolates of other *Marssonina* spp. (*Marssonina coronaria* and *Marssonina rosae*) were included, which also need to be further improved. Nevertheless, we had a wider detection range compared with the existing PCR-based and LAMP-based methods for *M. coronaria* detection [41,42,43], for which none of the other *Marssonina* species were tested in the specificity assay.

The simplicity of LAMP could also be described in the result visualization. Two formats in this study to judge the reaction results include visual inspection with HNB dye and agarose gel electrophoresis. The common intercalating dyes including calcein, SYBR green I, and HNB [24,28,48], as well as several other existing dyes, such as malachite green [49], Picogreen [50], Gelred [51], SYTO fluorescent [52], Evagreen [53], Goldview II [54], and berberine [55], were added into the reaction tube for visualization LAMP products. Positive reaction tube will exhibit color change or fluorescence, while for negative controls, this will not occur. Thus, unlike PCR, tedious procedure of gel electrophoresis is not needed. However, adding the dyes often requires the opening of the reaction tubes, therefore increasing the risk of carry-over contamination [56]. To avoid such contamination, we selected HNB dye as a visual indicator that we added to the LAMP reaction tube prior to amplification in our study. A positive reaction was indicated by a color change from violet to sky blue Figure 2A, Figure 3B, Figure 4B, Figure 5B, and Figure 6B). Moreover, it has been reported that the SYBR green I dye could inhibit LAMP reaction if the dye is added before isothermal incubation [57]. The pre-addition of 192 μM HNB into the LAMP reaction mixture enabled real-time monitoring with no inhibitory effect. Meanwhile, the results of the visual detection with HNB in this study were further verified by 2% agarose gel electrophoresis, as the result of positive tube after amplification will display a ladder-like pattern. The results obtained are perfectly in accordance with that of the visual detection with HNB Figure 2, Figure 3A,B, Figure 4A,B, Figure 5A,B, and Figure 6A,B).

In conclusion, the established LAMP assay in this study is rapid, sensitive, specific, simple, and low-cost, which is a promising diagnostic tool to replace conventional PCR-based methods for *M. brunnea* detection in both plant and environmental samples. This method has the potential for early diagnosis of *M. brunnea* and for greatly preventing poplar black spot disease in the field.

## 4. Materials and Methods

### 4.1. Design of LAMP Primers

The LAMP primers (Figure 1A) were designed on the basis of the rDNA *ITS* sequences of *Marssonina brunnea* f. sp. *multigermtubi* (Genbank accession no. KU508806) and *Marssonina brunnea* f. sp. *monogermtubi* (Genbank accession no. KM246324). Other related species (*M. mali*-EU520097, *M. rosae*-AY904059, and *M. coronariae*-JN587494) were retrieved from the GenBank database and aligned to identify highly conserved regions using the BioEdit software V7.2.0 [58] (Figure 1B). The LAMP primers were designed using the PRIMER EXPLORER V4 software program (http://primerexplorer.jp/e/) (Eiken Chemical Co., Ltd., Tokyo, Japan) and modified manually in order to increase the concentration of DNA produced during the LAMP reaction. A set of six primers were designed for the detection of *M. brunnea*, including two outer primers (forward primer F3 and backward primer B3) and two inner primers (forward inner primer FIP and backward inner primer BIP), which recognized six distinct regions of the target DNA, as well as two additional loop primers FL and BL, which can accelerate the LAMP reaction. Positions of primers within the nucleotide sequence of the *ITS* region are given in Figure 1. A BLASTn analysis of the target sequence was carried out to evaluate the specificity of the primers. Primers were synthesized and purified by Invitrogen Trading Shanghai Co., Ltd., and all sequences are listed in Table 1.

### 4.2. Fungal Materials and DNA Extraction

The strains used to evaluate the sensitivity and specificity of the primers are listed in Table 2. A total of 20 fungal isolates including 6 isolates of *M. brunnea* f. sp. *monogermtubi*, 6 isolates of *M. brunnea* f. sp. *multigermtubi*, 2 isolates of other reported *Marssonina* pathogens (*Marssonina coronaria* and *Marssonina rosae*), 3 isolates of common foliar fungal endophytes of poplar (*Alternaria* sp., *Botryosphaeria* sp., and *Pseudocercospora* sp.), and 3 isolates of other foliar pathogens of poplar (poplar leaf rust fungus *Melampsora larici-populina* Kleb., poplar leaf and shoot blight fungus *Venturia populina* (Vuill.) Fabr., and poplar spot anthracnose fungus *Elsinoë australis*) were used. Fungal cultures were maintained on potato dextrose agar (PDA; 200 g potato extract/L, 2% [*w/v*] glucose and 2% [*w/v*] agar) plates at 25 °C. Mycelium of each isolate was obtained by culturing in liquid potato dextrose broth (PDB; 200 g potato extract/L, 2% [*w/v*] glucose) at 25 °C for 3–5 days. Mycelia were harvested by filtration and frozen at −20 °C. Genomic DNA was extracted from mycelium with DNeasy Plant Mini Kit (Qiagen, Valencia, CA, USA) according to the manufacturer’s instructions. The concentration of DNA samples was determined by a NanoDrop ND-3300 fluorospectrometer (Thermo-Fisher Scientific, Wilmington, DE, USA). Extracted DNA was stored at −20 °C for further use. 

### 4.3. LAMP Reaction

The LAMP reaction was prepared in a 25 μL mixture containing 2.5 μL 10× ThermoPol Buffer, 4 μL MgSO_4_ (50 mM), 3.5 μL deoxynucleotide triphosphates (dNTPs) (10 mM), 2 μL each internal primer FIP and BIP (20 μM), 0.5 μL each external primer F3 and B3 (10 μM), 1 μL each loop primer FL and BL (10 μM), 4 μL betaine (5 M, Sigma-Aldrich, Saint Louis, MO, USA), 2 μL hydroxy naphthol blue HNB (2.4 mM, Aladdin, China), 1 μL *Bst* DNA polymerase (8 U/μL, New England BioLabs, Tokyo, Japan), and 1 μL of genomic DNA as the template. Negative controls containing an equivalent volume of nuclease-free water instead of DNA were included in each reaction. The final volume of each reaction was 25 μL. LAMP reactions were then optimized for amplification temperatures and incubation periods. The LAMP amplification mixtures were incubated at a constant temperature ranging from 60 to 67.5 °C for 1 hour, and then heated at 80 °C for 5 min to inactivate the *Bst* polymerase. Each treatment was replicated 3 times, and the experiment was repeated twice. To determine the optimal incubation periods, we mixed the LAMP amplification mixtures at the optimal temperature for 20 min to 1 h and then heated them at 80 °C for 5 min to stop the reaction. Subsequently, LAMP products were visualized directly by the naked eye. Samples that turned sky blue were considered to be *M. brunnea*-positive, while those that remained violet were considered to be *M. brunnea*-negative. The images of the LAMP tubes were captured with a Panasonic digital camera (Model: DMC-FZ28GK). After the visual assessment, the LAMP products were further verified by gel electrophoresis (2% agarose gel for 50 min at 80 V).

### 4.4. Conventional PCR Detection of M. brunnea

For conventional PCR, we used F3 and B3 primers to amplify the same specific region of rDNA *ITS* region. The PCR reaction mixture was composed of 1.0 μL DNA template, 12.5 μL PrimerSTAR Max Premix (2X; Takara, R045A), and 0.5 μL of each of the primers F3 (10 μM) and B3 (10 μM) (Table 1), adding nuclease-free H_2_O to a final volume of 25 μL. The thermal cycling program was 98 °C for 4 min; 31 cycles of 98 °C for 10 s, 56 °C for 5 s, and 72 °C for 5 s; and 72 °C for 5 min. After the amplification, PCR products were examined by 1.5% agarose gel electrophoresis for 30 min at 120 V, stained with ethidium bromide, and visualized by UV translumination. DNA ladder marker II (Tiangen, MD102, Beijing, China) was used for size references.

### 4.5. Assay of Specificity and Sensitivity in LAMP and PCR Methods

To evaluate the sensitivity of the LAMP and PCR assays, we tested DNA extracted from a representative isolate of *M. brunnea* XY-3 in 3 independent 10-fold dilution series ranging from 100 ng/μL to 1 fg/μL. DNA concentrations were estimated using a Nanodrop. In order to investigate the specificity of the LAMP and PCR assays, we tested DNA extracts from cultures of 12 isolates of *M. brunnea* (target species)*,* 2 isolates of other *Marssonina* spp. (non-target species most closely related in the genus), and 6 isolates of other fungal species (Table 2). The assays were evaluated on the basis of change in the color of HNB and the appearance of ladder-like pattern for LAMP and on 1.5% agarose gel electrophoresis for PCR. There were 3 replicates for each treatment, and the experiment was repeated twice.

### 4.6. LAMP and PCR Assays on the Artificially Inoculated Samples

To assess the ability of LAMP method to detect *M. brunnea* from infected samples, we prepared the artificially inoculated samples as described by Cheng et al. [59] with minor modifications. The pathogen-free branches of *P. tomentosa* Carr. and *Populus euramericana* cv. I-214 were cut out in winter and cultured in the greenhouse at 22 °C with a 12-h photoperiod. The cuttings were approximately 0.5–1 m high and produced 20 to 50 leaves after 12 weeks. A total of 8 fully expanded leaves from each poplar clone were collected, rinsed thoroughly with autoclaved water 5 to 6 times, and placed on sterilized 2% water agar culture plates with the abaxial surface of the leaf facing upward.

Conidia of *M. brunnea* f. sp. *multigermtubi* strain 214-4 and *M. brunnea* f. sp. *monogermtubi* strain QC2 were cultured in PDA medium for 10 days and suspended in sterile water, respectively. The conidia suspensions of strains 214-4 and QC2 were adjusted to 10^5^ spores/mL and sprayed onto the abaxial surface of the poplar leaves from clones *P. euramericana* cv. I-214 and *P. tomentosa* Carr.*,* respectively. The treated leaves were incubated in an illuminated incubator under 100% relative humidity (RH) at 22 °C with a 12-h photoperiod. After 3 days, visible disease symptoms began to develop. Symptomatic leaves infected by *M. brunnea* f. sp. *multigermtubi* strain 214-4 and *M. brunnea* f. sp. *monogermtubi* strain QC2 were harvested and then ground in liquid nitrogen immediately for DNA extraction, respectively. Pathogen-free leaves were excised from sterile tissue cultures of *P. tomentosa* Carr. clone (we could not generate tissue culture of *P. euramericana* cv. I-214 clone, despite numerous trials), then spray-inoculated with deionized water, which were used as a negative control. Genomic DNA (100 ng) extracted from the mycelium of *M. brunnea* strain XY-3 was used as a positive control. LAMP and PCR detection of *M. brunnea* in the inoculated poplar samples were performed using the above-described protocol and repeated at least twice per sample.

### 4.7. Detection of Poplar Black Spot Pathogen in the Field Samples by the LAMP and PCR Assays

To demonstrate the suitability of LAMP as a tool for the detection of *M. brunnea* in environmental samples, we collected a total of 16 leaves showing classical black spot symptoms from poplar trees (*P. tomentosa* Carr. and *P. euramericana* cv. I-214) located in the campus of Nanjing Forestry University (NJFU), where some of *M. brunnea* f. sp. *monogermtubi* and *M. brunnea* f. sp. *multigermtubi* strains we used in this study were respectively isolated from. Asymptomatic leaves collected from the *M. brunnea*-free site for several years at NJFU were used as a negative control (NC). For each sample, the purified genomic DNA was extracted by using a Plant DNA Mini Kit (Omega Biotek, China) according to the manufacturer’s protocol. The DNA concentration for each sample was determined by NanoDrop (ND-3300 fluorospectrometer, Thermo-Fisher Scientific, Wilmington, DE, USA). Both LAMP and PCR assays were carried out at least twice using 17 extracted DNAs as templates. A positive control (100 ng purified gDNA from mycelium of *M. brunnea* strain XY-3) and a no template (NT) control were included in each assay.

## Figures and Tables

**Table 1:** Sequences of the LAMP primers used for amplification of the target sequence in ITS region.

Primer Name	Primer Sequence (5′-3′)	Length/nt
F3 (forward outer primer)	CGCCAGAGGACCACAA	16
B3 (backward outer primer)	CCTTCGGAATGCCAAAGG	18
FIP (forward inner primer) (F1c + F2)	CCAGAACCAAGAGATCCGTTGTCCCGTGCCATGTCAGT	38
BIP (backward inner primer) (B1c + B2)	TGAAGAACGCAGCGAAATGCCGCAATGTGCGTTCAAAG	38
LF (forward loop primer)	ACTATTATATAGTACTCAGACGAC	24
LB (backward loop primer)	TGCAGAATTCAGTGAATCATCGA	23

**Table 2:** List of fungal strains used in this study.

Species	Isolate ID	Host	Origin	Order
*Marssonina brunnea* f. sp. *monogermtubi*	QC2	*Populus tomentosa* Carr.	Nanjing, Jiangsu	1
*Marssonina brunnea* f. sp. *monogermtubi*	QM2	*Populus tomentosa* Carr.	Nanjing, Jiangsu	2
*Marssonina brunnea* f. sp. *monogermtubi*	QM3	*Populus tomentosa* Carr.	Nanjing, Jiangsu	3
*Marssonina brunnea* f. sp. *monogermtubi*	QM6	*Populus tomentosa* Carr.	Nanjing, Jiangsu	4
*Marssonina brunnea* f. sp. *monogermtubi*	QM8	*Populus tomentosa* Carr.	Nanjing, Jiangsu	5
*Marssonina brunnea* f. sp. *monogermtubi*	QM15	*Populus tomentosa* Carr.	Nanjing, Jiangsu	6
*Marssonina brunnea* f. sp. *multigermtubi*	J1	*Populus × canadensis* Moench	Nanjing, Jiangsu	7
*Marssonina brunnea* f. sp. *multigermtubi*	J3	*Populus × canadensis* Moench	Nanjing, Jiangsu	8
*Marssonina brunnea* f. sp. *multigermtubi*	214-2	*Populus × euramericana* “I-214”	Nanjing, Jiangsu	9
*Marssonina brunnea* f. sp. *multigermtubi*	214-4	*Populus × euramericana* “I-214”	Nanjing, Jiangsu	10
*Marssonina brunnea* f. sp. *multigermtubi*	XY-1	*Populus simonii* Carr.	Xinmin, Liaoning	11
*Marssonina brunnea* f. sp. *multigermtubi*	XY-3	*Populus simonii* Carr.	Xinmin, Liaoning	12
*Marssonina coronaria* (Ell. & Davis) Davis	Mp-1	*Malus pumila* Mill.	Nanjing, Jiangsu	13
*Marssonina rosae* (Lib.) Fr.	Ms-3	*Rosa chinensis* Jacq.	Nanjing, Jiangsu	14
*Melampsora larici-populina* Kleb.	YL-1	*Populus × euramericana*	NJFU *	15
*Venturia populina* (Vuill.) Fabr.	T4	*Populus × euramericana* “I-214”	NJFU *	16
*Elsinoë australis*	NL-1	*Populus tomentosa* Carr. *Populus deltoides*	NJFU *	17
*Alternaria* sp.	As-1	*Populus × euramericana* “I-214” *Populus simonii* Carr. *Populus tomentosa* Carr.	NJFU *	18
*Botryosphaeria* sp.	Bs-1	*Populus × euramericana* “I-214” *Populus simonii* Carr. *Populus tomentosa* Carr.	NJFU *	19
*Pseudocercospora* sp.	Ps-1	*Populus × euramericana* “I-214” *Populus simonii* Carr. *Populus tomentosa* Carr.	NJFU *	20

* Nanjing Forestry University.

## Data Availability

All Data generated or analyzed are contained within the present article.
